# MHD hybrid nanofluid flow comprising the medication through a blood artery

**DOI:** 10.1038/s41598-021-91183-6

**Published:** 2021-06-02

**Authors:** Wajdi Alghamdi, Abdelaziz Alsubie, Poom Kumam, Anwar Saeed, Taza Gul

**Affiliations:** 1 Department of Information Technology, Faculty of Computing and Information Technology, King Abdulaziz University, Jeddah, 80261 Saudi Arabia; 2 Department of Basic Sciences, College of Science and Theoretical Studies, Saudi Electronic University, Riyadh, Saudi Arabia; 3 Center of Excellence in Theoretical and Computational Science (TaCS-CoE), Faculty of Science, King Mongkut’s University of Technology Thonburi (KMUTT), 126 Pracha Uthit Rd., Bang Mod, Thung Khru, Bangkok, 10140 Thailand; 4 Department of Medical Research, China Medical University Hospital, China Medical University, Taichung, 40402 Taiwan; 5 Department of Mathematics, City University of Science and Information Technology, Peshawar, Pakistan

**Keywords:** Engineering, Mathematics and computing, Nanoscience and technology

## Abstract

The current study focuses on the laminar flow of copper and copper oxide (${\text{Cu/blood}}$ and ${\text{Cu}} + {\text{CuO/blood}}$) hybrid nanoliquid, considering blood as a carrier fluid in a rectangular domain between two permeable channels. This study may manipulate for the purpose such as the drug delivery process, flow dynamic mechanism of the micro-circulatory system. In the proposed model, MHD and heat source/sink on the flow pattern have been studied. Furthermore, the sides of each channel are permeable, allowing the nanoliquid to escape, filter, squeezing and dilating with a fixed velocity. Appropriate transformations are incorporated to convert the governing partial differential equations and the boundary conditions suitable for computation. The elegant homotopy analysis method (HAM) is used to obtain analytic approximations for the resulting system of nonlinear differential equations. The features of flow characteristics such as velocity, and temperature profiles in response to the variations of the emerging parameters are simulated and examined with a physical explanation. The magnetic field plays a vital role in the blood flow and therefore the existing literature has been extending with the addition of magnetic field. Among the many outputs of the study, it is found that the pressure distribution decline with the accumulated values of the magnetic parameter at the center of the flow regime. The augmentation in the temperature distribution estimates the pH values and electric conductivity. Therefore, the ${\text{Cu}}\,\,{\text{and}}\,\,{\text{CuO}}$ hybrid nanofluids are used in this study for medication purposes. The magnetic field has an important role in the blood flow and therefore the extending study has been extending using the magnetic field. The heat emission/absorption term is added to the energy equation to maintain the homogeneous temperature for the blood flow. We expect that this work will provide efficient outputs for medical purposes such as drug delivery.

## Introduction

The nanoparticles are usually used in food items, medicines, nuclear reactors, agriculture, and so on. These fluids are prepared from the stable dispersion of the Nano sized particles in the base liquids such as water, ethylene glycol, Engine oil, blood and other liquids are known as nanofluids^16^. The dispersion of two or more materials in the same base fluid is known as the hybrid nanofluids. These fluids have many applications in the field of medical sciences and engineering. Most of the drugs are prepared in the form of hybrid nanofluids and blood is used as a testing base fluid to check the chemical reactions of the materials in the blood. The hybrid nanofluids are also used to enhance the thermal efficiency of the base liquids. For better blood circulation a normal viscosity and normal temperature are required to sustain the regularity in the transmission of blood. To upgrade the thermal characteristics of such fluid, nano-size particles are scattered in the base fluid, which enhances the thermal properties. Choi^1^ has presented a well-built concept to raise the heat transmission rate of such fluids. These fluids with enhanced thermophysical properties were termed nanofluids. The motivation for Choi’s pioneering work was the realization that base fluids with their low thermal conductivities are not efficient for modern heat transfer needs. The nano-sized particles added in nanoliquids comprise chemical, metals like $\left( {Cu,\,\,Al,\,Ag} \right)$, metal compound $\left( {SiC} \right)$, metal oxides (silica, alumina, and zirconia) nitrides $\left( {AlN,\,SiN} \right)$. As per nature and quantity, nanoliquids are classified (i) nanoliquids for the pollution purification purpose (ecological), (ii) nanofluid for heat transmission (iii) drug delivery (medical fields) (iv) pharmaceutical nanoliquids with numerous outcomes in several areas like oncology, immunology and cardiology^2^. Many research articles exist associated with the nanoliquid in which the enhancement of the heat transmission rate is elaborated with the standard carrier liquids^3–8^. The silver $Ag$ and aluminum *Al* have high reactivity as well as excellent thermal properties stability. Recently, a researcher has presented a new sort of nanoliquid to overcome the above limitation and enhances thermal characteristics by making a compound of different elements in a base fluid known as hybrid nanoliquids. This new emerging variety of nanoliquid has been utilized in several fields’ aspects as heat transmission, energy, medical, nano-electronics, and transportation in the naval system etc.

There are several of scientific articles related to hybrid nanoliquid are reported, which govern the enhancement in thermophysical property^9–13^. Ali and Sajid^14^ discussed the thermal conductivity of a hybrid nanoliquid through experimental and computational research. It the problem has been concluded that the rising trend of mass and energy fields increases the thermal conductivity. Furthermore, an appropriate choice of nanoparticles performs a mandatory role in hybrid nanoliquid stability. Rostami and Dinarvand^15^ studied the medical model such as liquid flow in the capillary drug delivery, blood oxygenation and particularly in the micro-circulatory system. They examined an electrically conducting hybrid ${\text{Cu}} + {\text{CuO}}/{\text{blood}}$ nanoliquid laminar flow passing through a horizontal surface. Later on, they rebound in another problem, the flow of hybrid $\left( {{\text{TiO}}_{2} + {\text{Ag}}/{\text{blood}}} \right)$ nanoliquid through a porous tube^16^. Their study provided a basic model for blood circulation in the respiratory system and drug delivery.

Ali et al.^17,18^ investigated the hybrid nanofluid peristaltic flow considering the TiO2–Cu and Cu–Al2O3 hybrid nanomaterials together with MHD and Jeffery fluid. The size and shape dependence effect of the heat transfer for cooling/heating purposes were studied by Saleem et al.^19^. The hybrid nanofluid tends to enhance the mass and heat transfer which have many applications in the pharmaceutical industries, preservation and the circulation of blood, and so on. Many researchers used the nanofluids/hybrid nanofluids in the verities of geometries^20–23^.

The liquid type substance, which circulates throughout the body and animals, is recognized as blood. Since the fluid in the present study is considered the purest blood, it is observed that the blood viscosity is not static according to the medical concept, and can control body pressure, hemoglobin ratio, and temperature and vessels/artery size as well. The large blood vessel is considered in this study. So, considering blood nature as suitable as a newtonian fluid. Politis et al.^24^ has implemented the numerical method by considering the Newtonian blood flow conditions and stable state, to investigate the several blood stream configurations. They concluded that the hemodynamic factors could be affected via different features. Koriko et al.^25^ has introduced the gold-blood concept over a horizontal upper side of a paraboloid. Later, Ijaz et al.^26^ scrutinized theoretically the CuO/blood transportation through the stenosis artery with the special characteristics of hemodynamics. The numerical calculation of blood in veins and arteries using non-Newtonian and Newtonian approaches with heat flux for biomedical sciences is simulated by Foong et al.^27^. The comparative investigation of copper/blood and copper-oxide/blood nanoliquids flow through the asymmetric channel is made by Bharathi and Prakash^28^.

The erythrocyte cell is an important magnetic factor, which is mainly possible that it will affect blood flow through arteries. Chen et al.^29^ has conducted a theoretical result by assuming magnetic effects on blood flow. The conducting fluids are mainly used for several applications in the biomedical field, including cellular separation, magnetic resonance imaging (MRI) or even as a contrast agent, as an active material quantification, treatment of tumor infected cells, MHD micropump and biomedical sensors^30^. Similarly, a magnetic role is also being examined for electromagnetic devices, cancer therapy, hydro-magnetic motors are also representing its importance. The existing and coming new development in MHD technologies has vast and real practical life uses^31^. Shamlooei and Sheikholeslami^32^ have studied the upshot of radiation on MHD nanoliquid by manipulating the finite element volume method. The MHD based hybrid nanofluid flows have been studied in^33–37^.

The study of the flow via contracting/enlarging the permeable object like channel or pipe has received the concentration of many researchers. They take part in many technical, biophysical and engineering issues. The role of such flow comprises heat exchanger, combustion chamber, and porous walled flow system and exhaust nozzles. Furthermore, such fluid flow in and animal bodies can be simulated as a flow through a contracting substance with pores allowing essential particles. Akinshilo^38^ presented the concept of nanoliquid flow passes through a permeable channel with contracting and expanding walls, which play an important role in shear stresses and heat transmission control. Ahmad et al.^39^ examined the carbon nanotubes based nanoliquid flows in squeezing and dilating rectangular porous wall channel. Several mathematicians and scientists have recently investigated such type flow models, which are elaborated through^40–42^.

The extension in the existing study^16^ has been pointed out as:

- The magnetic field has an important role in the blood flow and therefore the extending study^16^ has been extending using the magnetic field.
- Health acquired infections (IACs) are a main public health issue worldwide. Whereas Cuo nanofluid plays its important role as antimicrobial. ($CuO$) properties have a strong antimicrobial perspective and $CuO$ nanofluids are used in the Escherichia coli culture to assess their antibacterial potential. The enhancement in the temperature distribution evaluates the pH values and electric conductivity. Therefore, the $Cu\,\,{\text{and}}\,\,CuO$ hybrid nanofluids are used in this study for medication purposes.
- The variation in the temperature distribution is a regular phenomenon in the blood flow and in this study the energy equation is extended with the heat omission/absorption parameter.
- To tackle the solution, we applied the homotopy analysis method (HAM)^43–50^. The present study has a significant role in the physiological side. It is mostly applicable to the circulation of blood and drug delivery.

### Mathematical formulation

**Figure 1:**
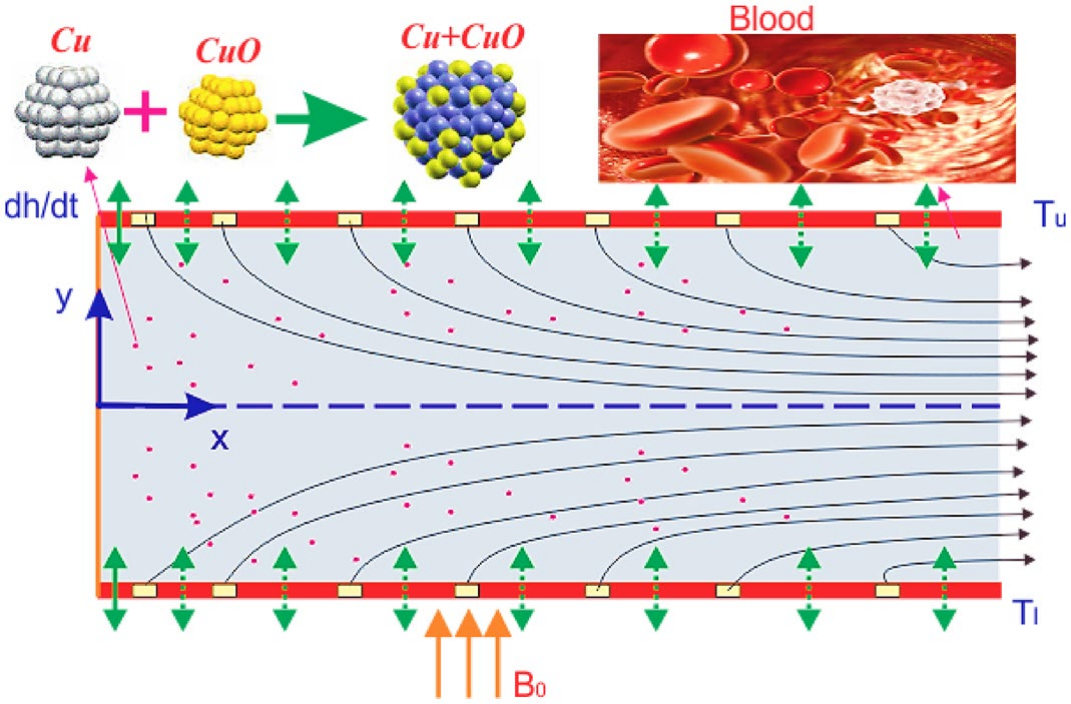
Geometry of the problem.

The copper oxide (${\text{Cu}} + {\text{CuO}}/{\text{blood}}$) hybrid nanoliquid flow with couple stress through a permeable channel in a rectangular domain is studied. In Fig. 1, the flow mechanism is shown. Where the velocity components *u* and *v* are designed in $x$ and *y*-directions for two-dimensional flow respectively. This model can be an appropriate model for heat transfer analysis/ hydrodynamic of arterial blood flow. The following presumptions will be considered via modeling the present flow problem:

- The hybrid nanofluid flow is isothermal and laminar.
- The length of the channel is assumed to be infinite and $2h\left( t \right)$ is the separation of the walls.
- One side of the channel is impermeable and flexible, while the other one is completely open for $Cu + CuO/{\text{blood}}$ nanoliquid flow.
- The permeable sides allow the exchange of fluid flow through dilation or contraction.
- Wall Sides are continuing contract and expand with the rate $h\left( t \right)$, which is dependent on time.
- The nano-size particles are assumed to be in thermal equilibrium.
- The heat source and sink, are considered in the energy equation.
- On behalf of the above flow presumptions, the governing equations can be rebound as^16,20,38^.
  1 $$\frac{{\partial \tilde{u}}}{\partial x} + \frac{{\partial \tilde{v}}}{\partial y} = 0,$$
  2 $$\rho_{hnf} \left( {\frac{{\partial \tilde{u}}}{\partial t} + \tilde{u}\frac{{\partial \tilde{u}}}{{\partial \tilde{x}}} + \tilde{v}\frac{{\partial \tilde{u}}}{{\partial \tilde{y}}}} \right) = - \frac{{\partial \tilde{P}}}{{\partial \tilde{x}}} + \mu_{hnf} \left[ {\frac{{\partial^{2} \tilde{u}}}{{\partial \tilde{x}^{2} }} + \frac{{\partial^{2} \tilde{u}}}{{\partial \tilde{y}^{2} }}} \right] - \sigma_{hnf} B_{0}^{2} \tilde{u},$$
  3 $$\rho_{hnf} \left( {\frac{{\partial \tilde{v}}}{\partial t} + \tilde{u}\frac{{\partial \tilde{v}}}{{\partial \tilde{x}}} + \tilde{v}\frac{{\partial \tilde{v}}}{{\partial \tilde{y}}}} \right) = - \frac{{\partial \tilde{P}}}{{\partial \tilde{y}}} + \mu_{hnf} \left[ {\frac{{\partial^{2} \tilde{v}}}{{\partial \tilde{x}^{2} }} + \frac{{\partial^{2} \tilde{v}}}{{\partial \tilde{y}^{2} }}} \right] - \sigma_{hnf} B_{0}^{2} \tilde{v},$$
  4 $$\left( {\rho C_{p} } \right)_{hnf} \left( {\frac{{\partial \tilde{T}}}{\partial t} + \tilde{u}\frac{{\partial \tilde{T}}}{{\partial \tilde{x}}} + \tilde{v}\frac{{\partial \tilde{T}}}{{\partial \tilde{y}}}} \right) = k_{hnf} \left[ {\frac{{\partial^{2} \tilde{T}}}{{\partial \tilde{x}^{2} }} + \frac{{\partial^{2} \tilde{T}}}{{\partial \tilde{y}^{2} }}} \right] + Q_{0} \left( {\tilde{T} - \tilde{T}_{0} } \right).$$

Here, $\eta$, $\rho_{hnf}$ and $\mu_{hnf}$ is density and dynamic viscosity, $\sigma_{{{\text{hnf}}}}$ and $k_{hnf}$ is the electrical and thermal conductivity of the hybrid nanofluid. $\left( {\rho C_{p} } \right)_{hnf}$ is the heat capacitance, $Q_{0}$ is a heat source/sink substrate, $\tilde{P}$ is the pressure and $h_{nf}$ manifest hybrid nanoliquid.

The boundary conditions are^16,38^:

5 $$\begin{gathered} \tilde{u} = 0,\,\,\,\,T = T_{l} ,\,\,\,\,\tilde{v} = - v_{l} = - \mathop {hA_{l} }\limits \,\,\,\,{\text{at }}\tilde{y} = - h\left( t \right), \hfill \\ \tilde{u} = 0,\,\,\,\,T = T_{u} ,\,\,\,\tilde{v} = - v_{u} = - \mathop h\limits A_{u} \,\,\,{\text{at}}\,\,\,\,\tilde{y} = \,\,\,\,\,h\left( t \right). \hfill \\ \end{gathered}$$

The blood is considered as the base fluid. In which, we have disseminated ${\text{Cu}}$ nanoparticles in the carrier fluid to synthesis mono-nanoliquid (${\text{Cu}}/{\text{blood}}$). Then ${\text{CuO}}$ nanoparticles are scattered in copper solution ${\text{Cu}}/{\text{blood}}$, to get the desired hybrid nanoliquid ${\text{Cu}} + {\text{CuO}}/{\text{blood}}$.

After putting

6 $$\chi = \left( {\frac{{\partial \tilde{v}}}{{\partial \tilde{x}}}} \right) - \left( {\frac{{\partial \tilde{u}}}{{\partial \tilde{y}}}} \right),$$

The cross differentiation has been used to neglect $\tilde{P}_{{\tilde{y}\tilde{x}}}$ from Eqs. (2, 3)**.**

7 $$\rho_{hnf} \left( {\frac{{\partial \tilde{\chi }}}{\partial t} + \tilde{u}\frac{{\partial \tilde{\chi }}}{{\partial \tilde{x}}} + \tilde{v}\frac{{\partial \tilde{\chi }}}{{\partial \tilde{y}}}} \right) = \mu_{hnf} \left[ {\frac{{\partial^{2} \tilde{\chi }}}{{\partial \tilde{x}^{2} }} + \frac{{\partial^{2} \tilde{\chi }}}{{\partial \tilde{y}^{2} }}} \right] - \sigma_{ hnf } B_{0}^{2} \frac{{\partial \tilde{\chi }}}{{\partial \tilde{y}}},$$

The obtained results are:

8 $$\rho_{hnf} \left( {\tilde{u}_{yt} + \tilde{u}\tilde{u}_{yx} + \tilde{v}\tilde{u}_{yy} } \right) = \mu_{hnf} \tilde{u}_{yyy} - \sigma_{ hnf } B_{0}^{2} \tilde{u}_{yy} .$$

### Transformation parameters

For unsteady blood flow the non-dimensional distance used as in^16^ and^38^:

9 $$yh = \eta .$$

Now, the similarity transformation are:

10 $$\,\tilde{u} = \frac{{\upsilon_{f} \tilde{x}\tilde{f}_{\eta } }}{{h^{2} }},\,\,\,\,\,\,\,\,\,\tilde{v} = \frac{{ - \upsilon_{f} \tilde{f}\left( {\eta ,t} \right)}}{h},\,\,\tilde{\psi } = \frac{{\upsilon_{f} \tilde{x}\tilde{f}\left( {\eta ,t} \right)}}{h},\,\,\theta \left( \eta \right) = \frac{\left( {\tilde{T} - \tilde{T}_{u} } \right)}{\left( {\tilde{T}_{l} - \tilde{T}_{u} } \right)}.$$

The physical conditions are depleted as

11 $$\begin{gathered} \tilde{f}_{\eta } = 0,\,\,\,\tilde{f} = {\text{Re}}_{l} ,\,\,\,{\text{Re}}_{l} = \frac{{h\mathop {h^{.}A_{l} }\limits }}{{\upsilon_{f} }}\,\,\,\,\,\,{\text{at}}\,\,\,\eta = - 1 \hfill \\ \tilde{f}_{\eta } = 0,\,\,\,\tilde{f} = {\text{Re}} ,\,\,\,{\text{Re}} = \frac{{h\mathop {h^{.}A_{u} }\limits }}{{\upsilon_{f} }}\,\,\,\,\,\,{\text{at}}\,\,\,\eta = 1 \hfill \\ \end{gathered}$$

Here, ${\text{Re}}$ explains the Reynolds number that is deliberating positive and adverse for injection and suction respectively.

Consequently, to dimensionless the velocity, we introduce:

12 $$u = \frac{{\tilde{u}}}{\mathop h\limits },\,\,\,\,v = \frac{{\tilde{v}}}{\mathop h\limits },\,\,\,x = \frac{{\tilde{x}}}{\mathop h\limits },\,\,\,f = \frac{{\tilde{f}}}{{\text{Re}}}.$$

The above-mentioned substrates in Eq. (10) are used to get the momentum equation as:

13 $$f^{\prime\prime\prime\prime} + \left( {1 - \phi_{2} } \right)^{2.5} \left( {1 - \phi_{1} } \right)^{2.5} \frac{{\rho_{hnf} }}{{\rho_{f} }}\left( {\left( {\eta f^{\prime\prime\prime} + 3f^{\prime\prime}} \right)\alpha + \left( {ff^{\prime\prime\prime} - f^{\prime}f^{\prime\prime}} \right)} \right){\text{Re}} - M^{2} f^{\prime\prime} = 0,$$

And

14 $$\begin{gathered} f = S,\,f^{\prime} = 0,\,\,\,\,\,{\text{at }}\eta = - 1, \hfill \\ f = 1,\,f^{\prime} = 0,\,\,\,\,\,\,{\text{at}}\,\,\eta = \,\,1. \hfill \\ \end{gathered}$$

Here $S = \left( {\frac{{A_{l} }}{{A_{u} }}} \right)$ defines the permeability parameter. On behalf of the above transformations the energy equation is transformed as:

15 $$\frac{{k_{hnf} }}{{k_{f} }}\theta^{\prime\prime} + \left( {\rho c_{p} } \right)_{hnf} \Pr \left( {\alpha \eta \theta^{\prime} + {\text{Re}} f\theta^{\prime}} \right) + Q\Pr \theta = 0,$$

And

16 $$\theta \left( { - 1} \right) = 1,\,\,\,\,\,\theta \left( 1 \right) = 0.$$

Here $\Pr$ convey Prandtl number and for the present studies, it is assumed as 21^16^. Also, it is supposed that $\upsilon = 2.8 \times 10^{ - 6} ({\text{m}}^{2} /{\text{s}})\,\,\,{\text{and }}\mu = 2.9 \times 10^{ - 3} ({\text{pa }}\,{\text{s)}}.$

The normal pressure is defined as:

17 $$\Delta P_{n} = - \frac{{\rho_{hnf} }}{{\rho_{f} }}\left[ {\frac{\alpha }{{\text{Re}}}\left( {\eta f} \right) + \left( {\frac{{f^{2} }}{2} - \frac{{f^{2} \left( 0 \right)}}{2}} \right) - \frac{{\mu_{hnf} }}{{\mu_{f}R_e }}\left( {f^{\prime} - f^{\prime}\left( 0 \right)} \right)} \right].$$

The wall deformation rate, heat source variable and Magnetic variable define as:

18 $$Q = \frac{{Q_{0} a^{2} }}{{\upsilon_{f} \left( {\rho C_{p} } \right)_{f} }},\,\,M^{2} = \frac{{\sigma_f B_{0}^{2} a^{2} }}{{\mu_{f} }}.$$

The simplified form of the drag force and rate of heat transfer for the hybrid nanofluid fluid at the both the lower and upper walls are defined as^16^:

19 $$\begin{gathered} {\text{Re}} (1 - \phi_{1} )^{2.5} (1 - \phi_{2} )^{2.5} Cf_{l} = f^{\prime\prime}( - 1),\,\,{\text{Re}} (1 - \phi_{1} )^{2.5} (1 - \phi_{2} )^{2.5} Cf_{l} = f^{\prime\prime}(1), \hfill \\ Nu_{l} = - \frac{{k_{hnf} }}{{k_{f} }}\theta^{\prime}( - 1),Nu_{u} = - \frac{{k_{hnf} }}{{k_{f} }}\theta^{\prime}(1). \hfill \\ \end{gathered}$$

### Solution procedure

The HAM opts to model equations for analytic results, which was presented by Liao^42,43^. To show the convergence, the sum of square residual error is achieved by using the BVP 2.0 package^44–50^. The initial approximation for velocity $f_{0}$ and temperature $\theta_{0}$ are given as

20 $$f_{0} \left( \eta \right) = \frac{3(1 - S)}{4}\left( {\eta - \frac{{\eta^{3} }}{3}} \right) + \frac{(1 - S)}{2},\,\,\theta_{0} \left( \eta \right) = \frac{(1 - \eta )}{2}.$$

The linear operators are presented as:

21 $${\rm L}_{\,f} (f) = f^{\prime\prime\prime\prime}{, }{\rm L}_{\,\theta } (\theta ) = \theta^{\prime\prime}.$$

The expand form of

22 $${\rm L}_{\,F} \left[ {\chi_{1} + \chi_{2} \eta + \chi_{3} \eta^{2} + \chi_{4} \eta^{3} } \right] = 0,{\rm L}_{\,G} \left[ {\chi_{3} + \chi_{4} \eta } \right] = 0,\,{\rm L}_{\,\,\Theta } \left[ {\chi_{5} + \chi_{6} \eta } \right] = 0. \,$$

The idea of the BVPh 2.0 package as defined by Liao^42^ applied to the modeled Eqs. (13, 15) with the initial and boundary conditions in Eqs. (14, 16) as:

23 $$\varepsilon_{m}^{f} = \frac{1}{n + 1}\sum\limits_{x = 1}^{n} {\left[ {N_{f} \left( {\sum\limits_{y = 1}^{m} {f(\eta ),} \,\sum\limits_{y = 1}^{m} {\theta (\eta ),} } \right)_{\eta = x\delta \eta } } \right]^{2} } ,$$

24 $$\varepsilon_{m}^{\theta } = \frac{1}{n + 1}\sum\limits_{x = 1}^{n} {\left[ {N_{\theta } \left( {\sum\limits_{y = 1}^{m} {f(\eta ),\sum\limits_{y = 1}^{m} {\theta (\eta )} } } \right)_{\eta = x\delta \eta } } \right]^{2} } ,$$

The total sum of the square residual is defined as Liao^41^$\varepsilon_{m}^{t} = \varepsilon_{m}^{F} + \varepsilon_{m}^{\Theta }$.

## Results and discussions

**Figure 2:**
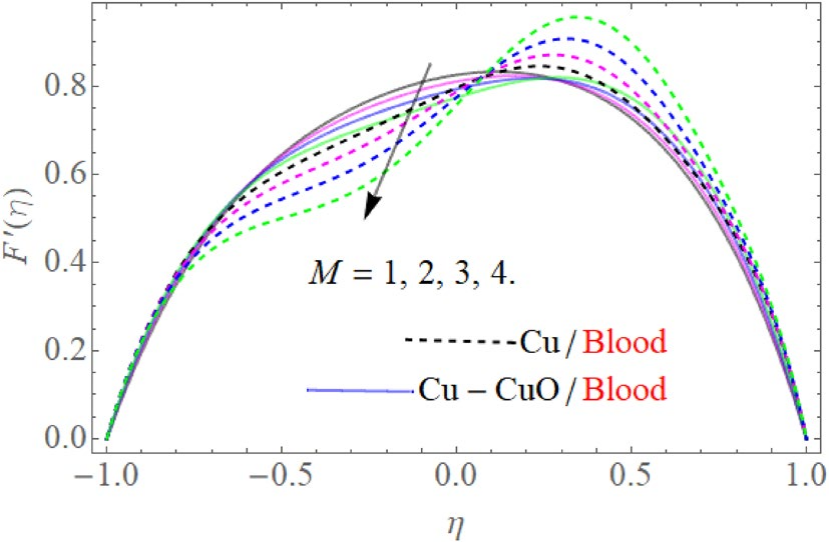
$$M$$ influence on $$F^{\prime}(\eta )$$.

**Figure 3:**
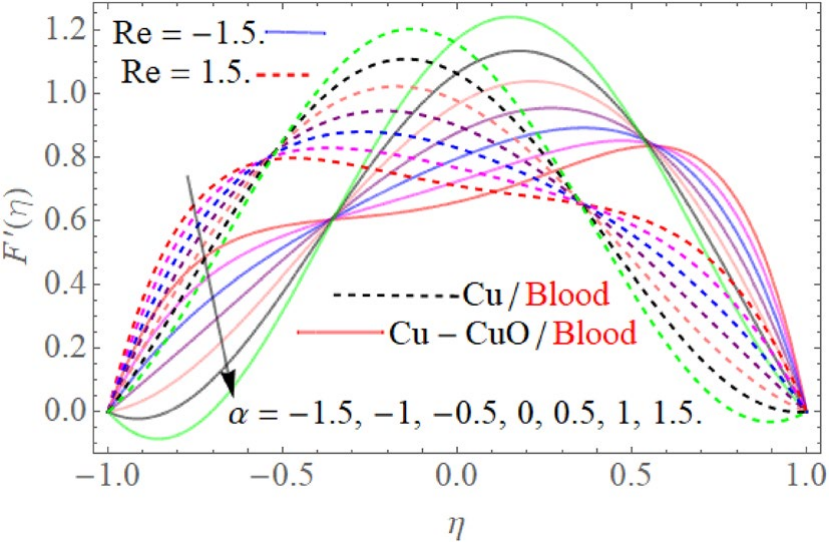
$\alpha$ influence on the $F^{\prime}(\eta )$.

**Figure 4:**
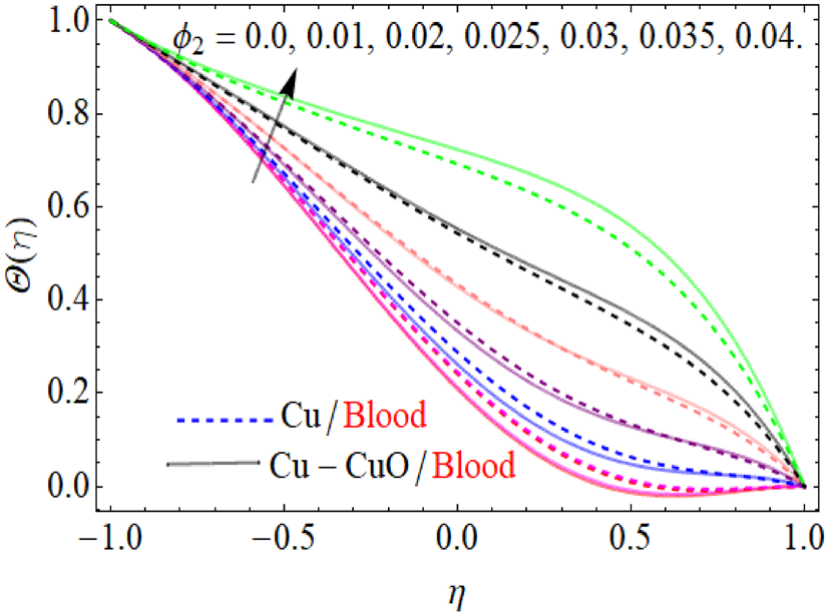
$\phi_{2}$ influence on the $\theta (\eta )$.

**Figure 5:**
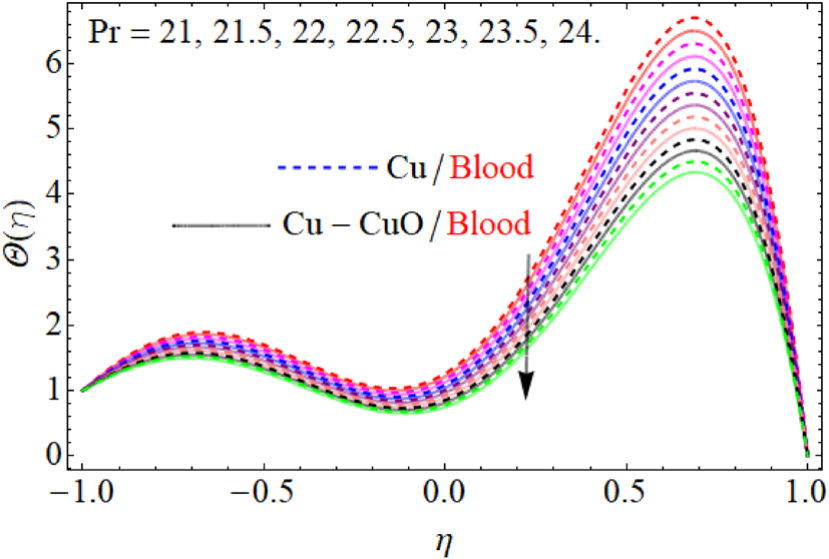
$$\Pr$$ influence on $$\theta (\eta )$$.

**Figure 6:**
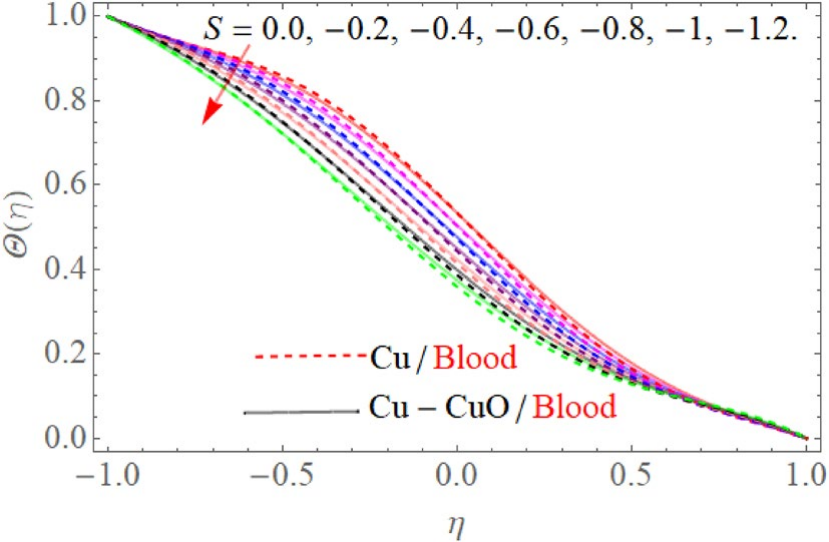
$$S$$ influence on $$\theta (\eta )$$.

**Figure 7:**
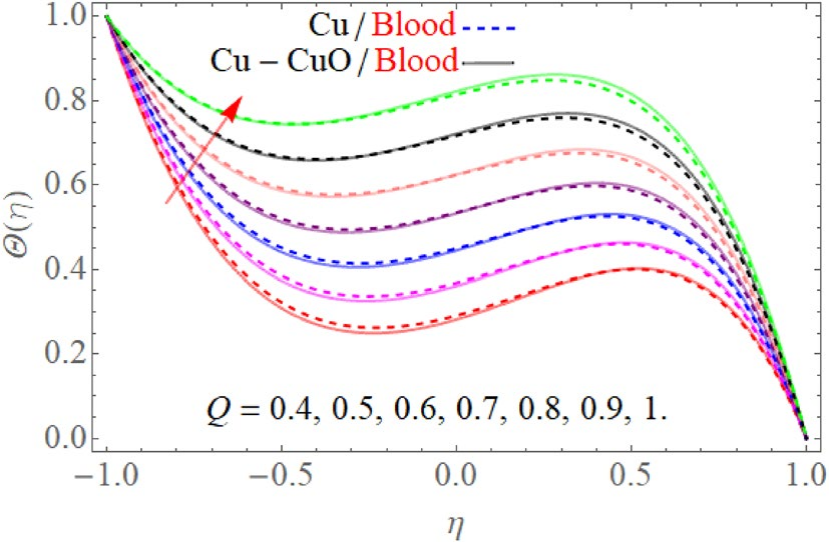
$$Q > 0$$ influence on $$\theta (\eta )$$.

**Figure 8:**
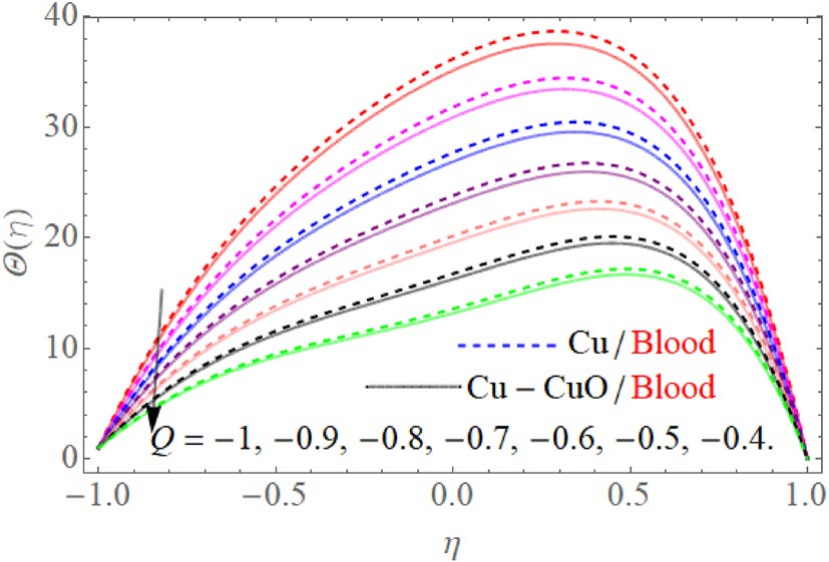
$$Q < 0$$ influence on $$\theta (\eta )$$.

We elaborate on the physical detail of interest constraints relevant to the present blood flow model through tables and figures. The outputs of these variables present in literature have been published by several scholars based on experimental and theoretical work. The numerical value of the Prandtl number $\Pr$ is settled, fixed 21 for blood^16^. The figures are built through HAM and their significance is revealed through Figs. 2, 3, 4, 5, 6, 7 and 8 via using thermo-physical properties. The physical mechanism of the problem is given in Fig. 1. The influence of the $f^{\prime}\left( \eta \right)$ against different physical constraints like magnetic field *M*, suction/injection $\alpha$ and *K* has been observed in Figs. 2, 3 and 4 respectively. The upshots of the magnetic strength $M$ on velocity profile $f^{\prime}\left( \eta \right)$ is displayed in Fig. 2, by considering $\Pr = 21,\,S = - 0.2,\,Q = 1,\,{\text{Re}} = 2.5,\,\,\phi_{2} = \phi_{1} = 0.01.$ The rising credit of $M$ reduces the velocity profile near the channel center. Thus, the magnetic strength produces the force in the opposite direction. Such force (Lorentz force) provides resistance to the flow field. This force can be more intensified by enhancing the strength of the magnetic field, which causes the nanoliquid flow to reduce inside the channel. It also inversely affects the viscosity of blood.

Figure 3 revealed the nature of the velocity profile $f^{\prime}\left( \eta \right)$ versus the suction/injection parameter. We perceived that, by rising credit of parameter $\alpha$, the velocity profile $f^{\prime}\left( \eta \right)$ gradually reduces, but an opposite seen has been observed as we move towards the hemodynamic boundary layer center. Consequently, it can be noticed that in both situations (injection/suction), when the channel sides expand $\alpha > 0$, it improves the velocity $f^{\prime}\left( \eta \right)$. Physically, when channel walls expand, space is generated near the channel wall. So, to cover that palace the nanoliquid flow move towards the center of the channel walls. On the other hand, when channel walls contract by taking $\alpha < 0$. The absolute credit of $\alpha$ leads to an enhancement in the flow field near the side or walls. As a consequence of this, the squeezing, as well as reduction in the flow field, was observed to keep momentum conservation maintain.

The upshots of volume fraction $\phi_{2}$( $CuO$) on temperature $\theta (\eta )$ are depicted in Fig. 4. It can be perceived that the fluid $\theta (\eta )$ is rising for the positive increment of copper-oxide volume fraction $\phi_{2}$ ($CuO/{\text{blood}}$). Physically, the increasing credit of $\phi_{2}$ reduces the specific heat capacity of nanoliquid, because the specific heat capacity of $CuO$ is less than blood. Furthermore, it also enhances the thermal expansion of nanoliquid, that’s why the fluid temperature enhances with the increases of $\phi_{2}$.

Figure 5 expresses the consequences of the Prandtl number $\Pr$ on temperature $\theta (\eta )$ profile. Physically, higher Prandtl fluid reduces the thermal boundary layer as well as has less thermal diffusivity, that’s why temperature decline with the effect of the Prandtl number $\Pr$*.* The effect of $S$ in the case of contraction and injection as well as dilation and injection is drawn in Fig. 6. So, the temperature profile enhances with the rising credit of |S|. However, upper wall temperature is slightly affected by the variation |S|. Because, $T_{l} > T_{u}$, that’s why the upper wall is less affected due to injection, the fluid near the wall carries more heat as compared to the upper wall. Figures 7 and 8 illustrate the trend of $\theta (\eta )$ versus the variation of heat absorption $\left( {Q < 0} \right)$ and heat generation $\left( {Q > 0} \right)$. Variation in $\left( {Q > 0} \right)$ bringing an enhancement in the $\theta (\eta )$ field, because the generated energy raises the thermal gradient. That’s why the growing value of $\left( {Q > 0} \right)$ term augmenting the temperature distribution. Further, the behavior of $\theta (\eta )$ profile against the negative value of $Q$ is illustrated in Fig. 8. The negative values of $Q$ decline the temperature profile. Because the heat absorption factor absorbs the heat from the channel surface which reduces the fluid temperature.

**Figure 9:**
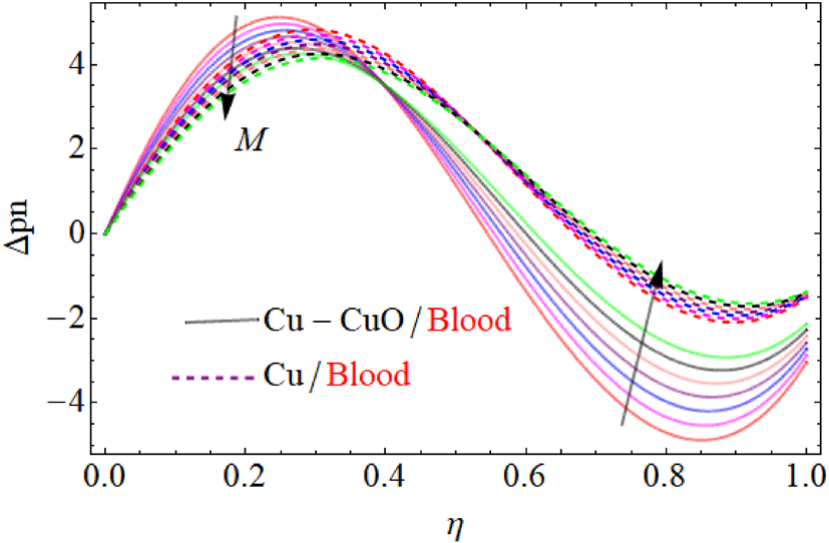
$$M$$ influence on $$\Delta pn$$.

**Figure 10:**
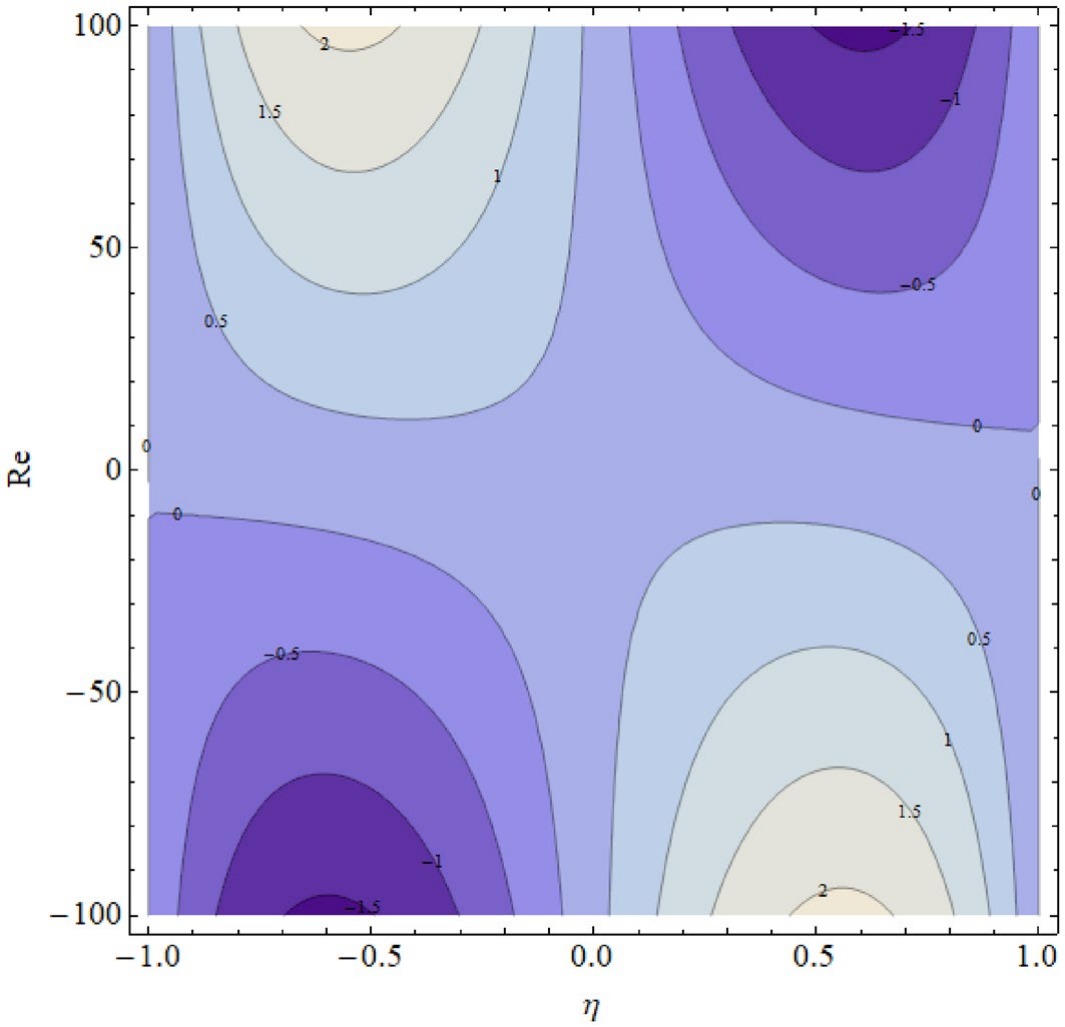
Contour upshot due to the variation in ${\text{Re}}$.

**Figure 11:**
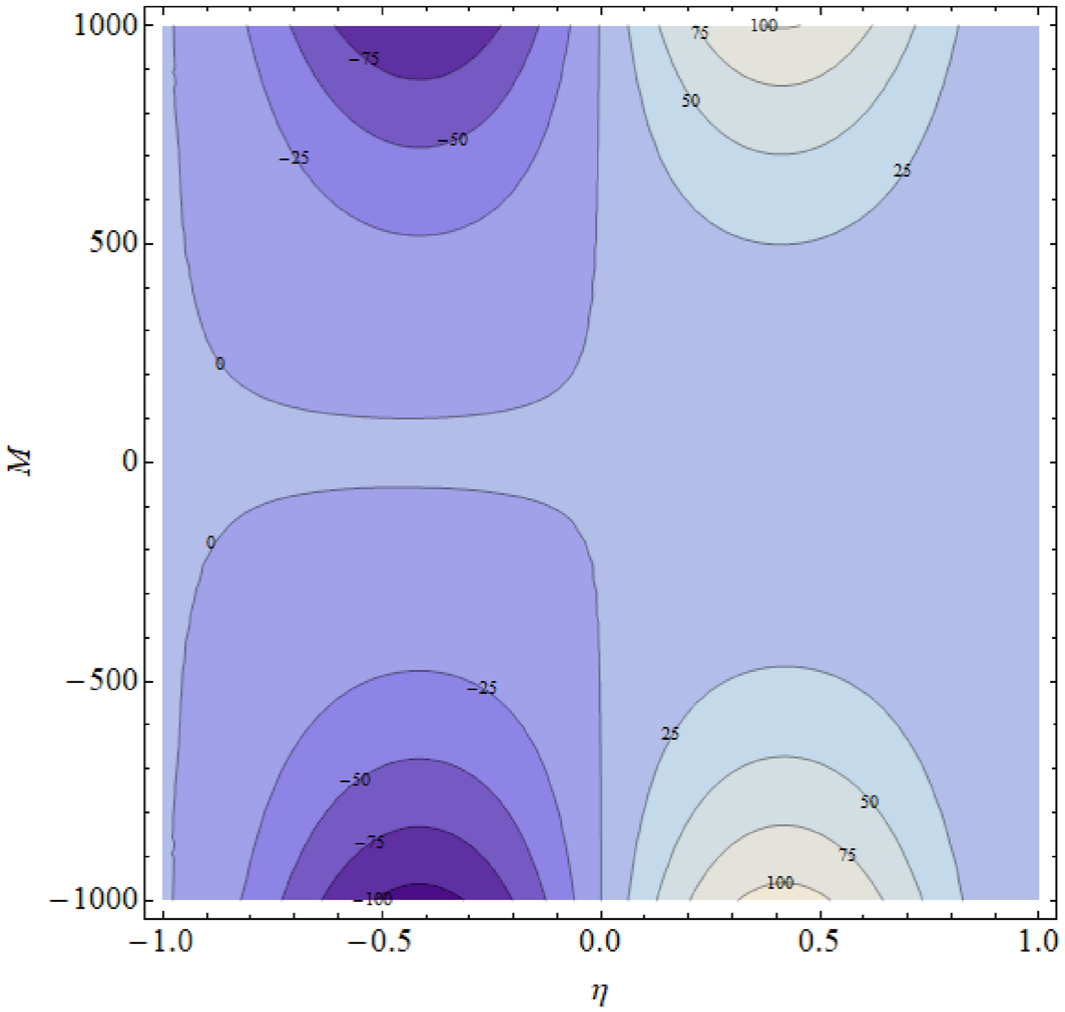
Contour upshot due to the variation in $M$.

**Figure 12:**
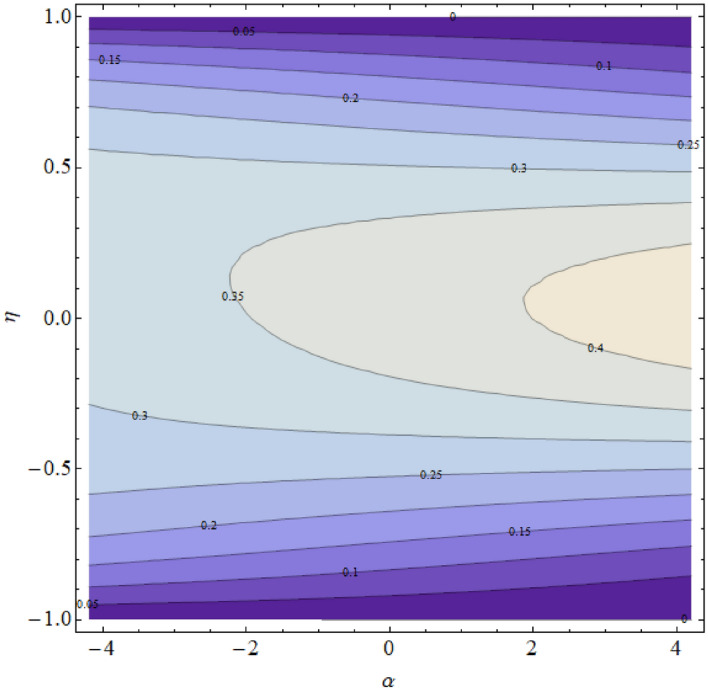
Contour upshot due to the variation in $\alpha$.

The influence of the magnetic parameter $M$ has been displayed in Fig. 9 respectively. The pressure distribution decline near the center of the two walls with the increasing values of these parameters and boost up away from the center. In fact, during fluid flow in a channel, the pressure distribution is strongest in the center; therefore, the parameter effect is mainly counted at the center of the channel. Also, the rising values of these parameters provide more resistance to reduce the fluid motion, and consequently the pressure distribution decline. The contour figures under the impact of ${\text{Re}} ,M,\alpha$ are shown in Figs. 10, 11 and 12.

Table 1. displays the thermophysical properties of copper $Cu$ and copper oxide, $CuO$ hybrid nanoliquid fluid. Tables 2 and 3 scrutinized the flow model for $Cu/{\text{blood}}$ and $CuO/{\text{blood}}$ respectively. Table 4 illustrates the Skin friction at lower and upper cases versus different values of physical constraints such as ${\text{Re}} ,M$**.** The increasing value of these constraints boosts up the skin friction at both walls. The skin friction using hybrid nanofluid is more effective. In fact, these parameters enhance the resistive force against the fluid flow, and hybrid nanofluids are comparatively denser than common fluids. The influence of the physical constraints over the heat transfer rate has been detected in Table 5. The rise in the value of the volume fractions $\phi_{1} ,\phi_{2}$ improves the heat transfer rate. It has been detected that the hybrid nanofluids are more efficient to enhance the heat transfer rate as compared to conventional fluids. The Prandtl number $\Pr$ for the blood is initially 21 and increasing the value of $\Pr$ decline the heat transfer rate. This effect is also very efficient using the hybrid nanofluid as displayed in Table 5. The parameter $Q > 0$ raises the heat transfer rate and the effect is more influential using the hybrid nanofluid. The comparison of the present work with the existing literature^16^ using the HAM technique has been analyzed in Table 6. The closed agreement of the present study with the existing study authenticates the obtained results.

**Table 1:** Thermo physical characteristics of blood, ${\text{CuO}}\,\,{\text{and}}\,\,{\text{Cu}}$^15,27^.

Thermo-physical prop	$c_{p}$	$\rho$	$k$	$\beta \times 10^{5}$
Blood	3594	1063	0.492	0.18
${\text{Cu}}$	385	8933	400	1.67
${\text{CuO}}$	533	6500	17.65	1.8

**Table 2:** The following model is considered for ${\text{Cu}}/{\text{blood}}$ nanoliquid^15,27^.

Density	$\frac{{\rho_{nf} }}{{\rho_{f} }} = \left( {1 - \phi_{1} } \right) + \phi_{1} \, \frac{{\rho_{Cu} }}{{\rho_{f} }}$
Viscosity	$\frac{{\mu_{nf} }}{{\mu_{f} }} = \frac{1}{{\left( {1 - \phi_{1} } \right)^{2.5} }}$
Specific heat	$\left( {\rho c_{p} } \right)_{nf} = \left[ {\phi_{1} \, \left( {\frac{{\left( {\rho \, cp} \right){}_{Cu}}}{{\left( {\rho \, cp} \right)_{f} }}} \right) + \left( {1 - \phi_{1} } \right)} \right]\left( {\rho cp} \right)_{f}$
Thermal conductivity	$k_{nf} \, = k_{f} \left\{ {\frac{{k_{Cu} - 2\phi_{1} (k_{f} - k_{Cu} ) + 2k_{f} }}{{k_{Cu} + 2\phi_{1} (k_{f} - k_{Cu} ) + 2k_{f} }}} \right\}$

**Table 3:** The following model is considered for ${\text{Cu}} + {\text{CuO}}/{\text{blood}}$ hybrid nanoliquid^15,27^.

Density	$\frac{{\rho_{hnf} }}{{\rho_{f} }} = \left( {1 - \phi_{2} } \right)\left[ {1 - \left( {1 - \frac{{\rho_{Cu} }}{{\rho_{f} }}} \right)\phi_{1} \, } \right] + \phi_{2} \, \frac{{\rho_{CuO} }}{{\rho_{f} }}$
Viscosity	$\frac{{\mu_{hnf} }}{{\mu_{f} }} = \frac{1}{{\left( {1 - \phi_{1} } \right)^{2.5} \left( {1 - \phi_{2} } \right)^{2.5} }}$
Specific heat	$\left( {\rho \, c_{p} } \right)_{hnf} = \left[ {\left( {1 - \phi_{1} } \right)\left( {1 - \phi_{2} } \right) + \phi_{2} \, \left( {\frac{{\left( {\rho \, cp} \right){}_{CuO}}}{{\left( {\rho \, cp} \right)_{f} }}} \right) + \phi_{1} \, \left( {\frac{{\left( {\rho \, cp} \right){}_{Cu}}}{{\left( {\rho \, cp} \right)_{f} }}} \right)} \right]\left( {\rho \, cp} \right)_{f}$
Thermal conductivity	$k_{nf} \, = k_{f} \left\{ {\frac{{k_{Cu} - 2\phi_{1} (k_{f} - k_{Cu} ) + 2k_{f} }}{{k_{Cu} - 2\phi_{1} (k_{f} - k_{Cu} ) + 2k_{f} }}} \right\}\left\{ {\frac{{k_{CuO} - 2\phi_{2} (k_{nf} - k_{CuO} ) + 2k_{nf} }}{{k_{CuO} + 2\phi_{2} (k_{nf} - k_{CuO} ) + 2k_{nf} }}} \right\}$

**Table 4:** Skin friction at lower and upper cases versus different values of physical constraints.

${\text{Re}}$	$M$	$\frac{1}{{(1 - \phi_{2} )^{2.5} }}f^{\prime}(1)$ Nano	$\frac{1}{{(1 - \phi_{2} )^{2.5} }}f^{\prime}( - 1)$ Nano	$\frac{1}{{(1 - \phi_{1} )^{2.5} }}f^{\prime}(1)$ Hybrid	$\frac{1}{{(1 - \phi_{1} )^{2.5} }}f^{\prime}( - 1)$ Hybrid
1	3	0.206163	2.724668	1.31997	3.59282
2		0.252397	3.06905	1.56111	3.18395
3		0.387489	3.36951	1.76290	4.31076
	4	0.502814	4.08795	1.93997	5.80282
	5	0.528333	4.23457	1.97234	5.98209

**Table 5:** Nusselt number for lower and upper case versus different values of physical constraints**.**

$\phi_{1}$	$\phi_{2}$	$\Pr$	$Q$	$- (\frac{{k_{\eta f} }}{{k_{f} }})\theta ^{\prime}(1)$ Nano	$- (\frac{{k_{\eta f} }}{{k_{f} }})\theta ^{\prime}( - 1)$ Nano	$- (\frac{{k_{\eta f} }}{{k_{f} }})\theta ^{\prime}(1)$ Hybrid	$- (\frac{{k_{\eta f} }}{{k_{f} }})\theta ^{\prime}( - 1)$ Hybrid
0.0	0.0	21	0.1	4.22538	1.03645	4.22538	1.03645
0.01				9.18698	5.9991	10.74611	8.1287
0.02				14.0894	9.56781	15.86296	14.6645
	0.01			6.88769	4.894510	7.98276	6.34980
	0.02			11.5498	10.01128	13.9677	13.9898
		22		9.68015	7.34298	8.6648	6.38761
		23		8.8887	6.6729	7.0991	5.9854
			0.2	5.05618	2.71365	3.16881	4.98765
			0.3	6.80572	6.99019	7.9853	8.5701

**Table 6:** Comparison of the present study and existing literature^16^ considering common parameters $\phi_{1} = \phi_{2} = M = Q = 0,{\text{Re}} = 1,\Pr = 21.$

$f^{\prime}(1)$ ^16^	$f^{\prime}( - 1)$ ^16^	$f^{\prime}(1)$ Present	$f^{\prime}( - 1)$ Present	$- \theta ^{\prime}(1)$ ^16^	$- \theta ^{\prime}( - 1)$ ^16^	$- \theta ^{\prime}(1)$ Present	$- \theta ^{\prime}( - 1)$ Present
0.205241	2.713240	0.20432001	2.714102110	4.214271	1.0243651	4.2132021	1.02320121

## Conclusion

The flow of the hybrid nanofluid flow through a deformable asymmetric channel of permeable walls has been analyzed. The ${\text{Cu}}$ and ${\text{CuO}}$ nanoparticles are suspended in the blood. The heat transfer rate and skin friction for the proposed model are calculated numerically and discussed. The HAM technique has been used for the solution of the problem. The obtained outcomes are pointed out as:

- The pressure distribution decline with the accumulated values of the magnetic parameter at the center of the flow regime. The variation in these parameters can play an important role in controlling the pressure distribution during the drug delivery to sustain the stability of blood flow in the vessels.
- It has been noticed that in both situations (injection/suction), when the channel sides expand $\alpha > 0$, it improves the blood velocity. On the other hand, when channel walls contract by taking $\alpha < 0$. As a consequence of this, the squeezing, as well as reduction in the flow field, was observed to keep momentum conservation maintain.
- The ${\text{CuO}}$ nanofluid plays its important role as antimicrobial. (${\text{CuO}}$) properties have a strong antimicrobial perspective and ${\text{CuO}}$ nanofluids are used in the Escherichia coli culture to assess their antibacterial potential. The focus has been given to the mentioned applications using the variation in the nanoparticle volume fraction.
- The enhancement in the temperature distribution evaluates the pH values and electric conductivity and therefore, the ${\text{Cu}}\,\,{\text{and}}\,\,{\text{CuO}}$ hybrid nanofluids are used in this study for medication purposes.
- The variation in the temperature distribution is a regular phenomenon in the blood flow and in this study, the heat omission/absorption parameter plays the role to maintain a reasonable temperature for the blood supply.
- The outcomes show that the hybrid nanofluid (${\text{Cu}} + {\text{CuO/blood}}$) is more effective for the thermal analysis and drug deliveries in the contracting expanding channels.

## Data Availability

The data that support the findings of this study are available from the corresponding author upon reasonable request.
